# Microbial Gene Abundance and Expression Patterns across a River to Ocean Salinity Gradient

**DOI:** 10.1371/journal.pone.0140578

**Published:** 2015-11-04

**Authors:** Caroline S. Fortunato, Byron C. Crump

**Affiliations:** 1 Josephine Bay Paul Center, Marine Biological Laboratory, Woods Hole, Massachusetts, United States of America; 2 College of Earth, Oceans, and Atmospheric Sciences, Oregon State University, Corvallis, Oregon, United States of America; Laval University, CANADA

## Abstract

Microbial communities mediate the biogeochemical cycles that drive ecosystems, and it is important to understand how these communities are affected by changing environmental conditions, especially in complex coastal zones. As fresh and marine waters mix in estuaries and river plumes, the salinity, temperature, and nutrient gradients that are generated strongly influence bacterioplankton community structure, yet, a parallel change in functional diversity has not been described. Metagenomic and metatranscriptomic analyses were conducted on five water samples spanning the salinity gradient of the Columbia River coastal margin, including river, estuary, plume, and ocean, in August 2010. Samples were pre-filtered through 3 μm filters and collected on 0.2 μm filters, thus results were focused on changes among free-living microbial communities. Results from metagenomic 16S rRNA sequences showed taxonomically distinct bacterial communities in river, estuary, and coastal ocean. Despite the strong salinity gradient observed over sampling locations (0 to 33), the functional gene profiles in the metagenomes were very similar from river to ocean with an average similarity of 82%. The metatranscriptomes, however, had an average similarity of 31%. Although differences were few among the metagenomes, we observed a change from river to ocean in the abundance of genes encoding for catabolic pathways, osmoregulators, and metal transporters. Additionally, genes specifying both bacterial oxygenic and anoxygenic photosynthesis were abundant and expressed in the estuary and plume. Denitrification genes were found throughout the Columbia River coastal margin, and most highly expressed in the estuary. Across a river to ocean gradient, the free-living microbial community followed three different patterns of diversity: 1) the taxonomy of the community changed strongly with salinity, 2) metabolic potential was highly similar across samples, with few differences in functional gene abundance from river to ocean, and 3) gene expression was highly variable and generally was independent of changes in salinity.

## Introduction

As fresh and marine waters mix in estuaries and river plumes, salinity, temperature, and nutrient gradients develop spatially from river to ocean. Globally over a broad range of environments, including ocean, freshwaters, and soils, salinity strongly correlates with changes in bacterial community composition [1] and many studies show that bacterioplankton community composition changes with salinity [2–6]. Specifically in the Columbia River coastal margin, previous work identified taxonomically distinct bacterioplankton communities in fresh, estuarine, plume, and surface ocean waters [7, 8]. Although communities have been shown to change taxonomically, little is known about river-to-ocean patterns in functional diversity.

A difference in functional processes operating within members of microbial communities have been characterized across many gradients, including light, nutrients, and oxygen. For example, Hewson et al. [9] showed strikingly different gene expression patterns in surface and bottom waters of a stratified estuary, with dramatic changes in microbial gene expression associated with the formation of anoxic bottom waters. In a marine oxygen minimum zone, Stewart et al. [10] compared metagenomic and metatranscriptomic data among samples collected across an oxygen gradient and observed differences in gene abundance and expression, as nitrogen cycling processes changed from oxidative to reductive and the community changed from nitrifying taxa to taxa associated with anammox and denitrification. These studies demonstrated the metabolic responsiveness of microbial communities to environmental gradients, and revealed potentially important ecological process and microbial community dynamics in biogeochemically active environments.

In this study we focused on the environmental gradients that occur from river to ocean. River to ocean environments are biogeochemically dynamic and microbial communities encounter changes in salinity, turbidity, and concentrations of nutrients and particulate and dissolved organic carbon as water flows from fresh to salt. To date there have been few studies describing gene abundance and no studies describing gene expression across the environmental gradients of a river to ocean continuum. Recently, Dupont et al. [11] described differences in the central metabolism of microbes across the salinity gradient of the Baltic Sea, including significant differences in the abundance of genes associated with respiration, glycolysis, and transport systems. Comparisons of freshwater lake and marine metagenomes also showed differences in functional gene abundance among microbial communities, specifically in nutrient transport, carbohydrate metabolism, and osmoregulation [12, 13]. Ghai et al. [14] compared river and ocean metagenomes and showed higher abundances of genes related to heterotrophic carbon processing, especially processing of allochthonous carbon input in the Amazon River, when compared to marine systems.

In this study, we focused on the analysis of gene abundance and expression patterns across the salinity gradient of the Columbia River coastal margin. The Columbia River is the second largest river in the United States with a mean annual discharge of 7300 m^3^s^-1^ and is the largest source of freshwater to the northeast Pacific Ocean [15, 16]. The river delivers allochthonous organic carbon as well as iron and silica to the estuary and the coastal ocean. [17, 18]. The significant release of nutrient rich freshwater has a large impact on the chemical, physical, and biological characteristics of the river plume and the Oregon and Washington coasts [18]. A previous study of microbial communities in the Columbia River coastal margin using microarray data demonstrated strong seasonal shifts in gene expression patterns, while spatial differences in expression were most apparent in spring and very limited in August [19]. This contrasted with a study of microbial community composition in the same system based on 16S rRNA genes, which showed that spatial variability was much greater than seasonal variability [7]. More recent work on the system involved analysis of twelve size-fractionated metagenomes from four samples collected in August 2007 across the Columbia River coastal margin from deep shelf waters, hypoxic waters, the river plume, and estuary. Analysis of these samples showed evidence of anaerobic micro-niches within particles and photoheterotrophy in the plume and estuary samples [20]. The samples analyzed in the current study focus on the free-living microbial community across changing salinities to gain a better understanding of how the functional profiles of these communities vary from fresh to marine waters.

Building on patterns of community structure and previous studies of the coastal margin, the addition of both gene abundance and expression data creates a link between community structure and function and provides information to explore how and why specific populations are distributed from river to ocean. We hypothesized that the metabolic potential of the free-living microbial community would change dramatically from river to ocean, but that taxonomically distinct populations would perform similar metabolic activities regardless of the salinity of the water. Our results, however, showed the opposite. The metabolic potential, assessed with metagenomics, varied only slightly across the salinity gradient despite large taxonomic differences. In contrast, metabolic activity, assessed with metatranscriptomics, showed little relationship with salinity and was highly variable, with each metatranscriptome dominated by a handful of different functional genes.

## Methods

This study required no specific permissions for sampling locations or activities. Sampling was carried out in the main channel of the river and estuary or directly off the coast and was not completed on private or protected lands. In addition, sampling was restricted to collection of river, estuary, or ocean water only and did not involve collection of or interaction with protected or endangered species.

**Fig 1:**
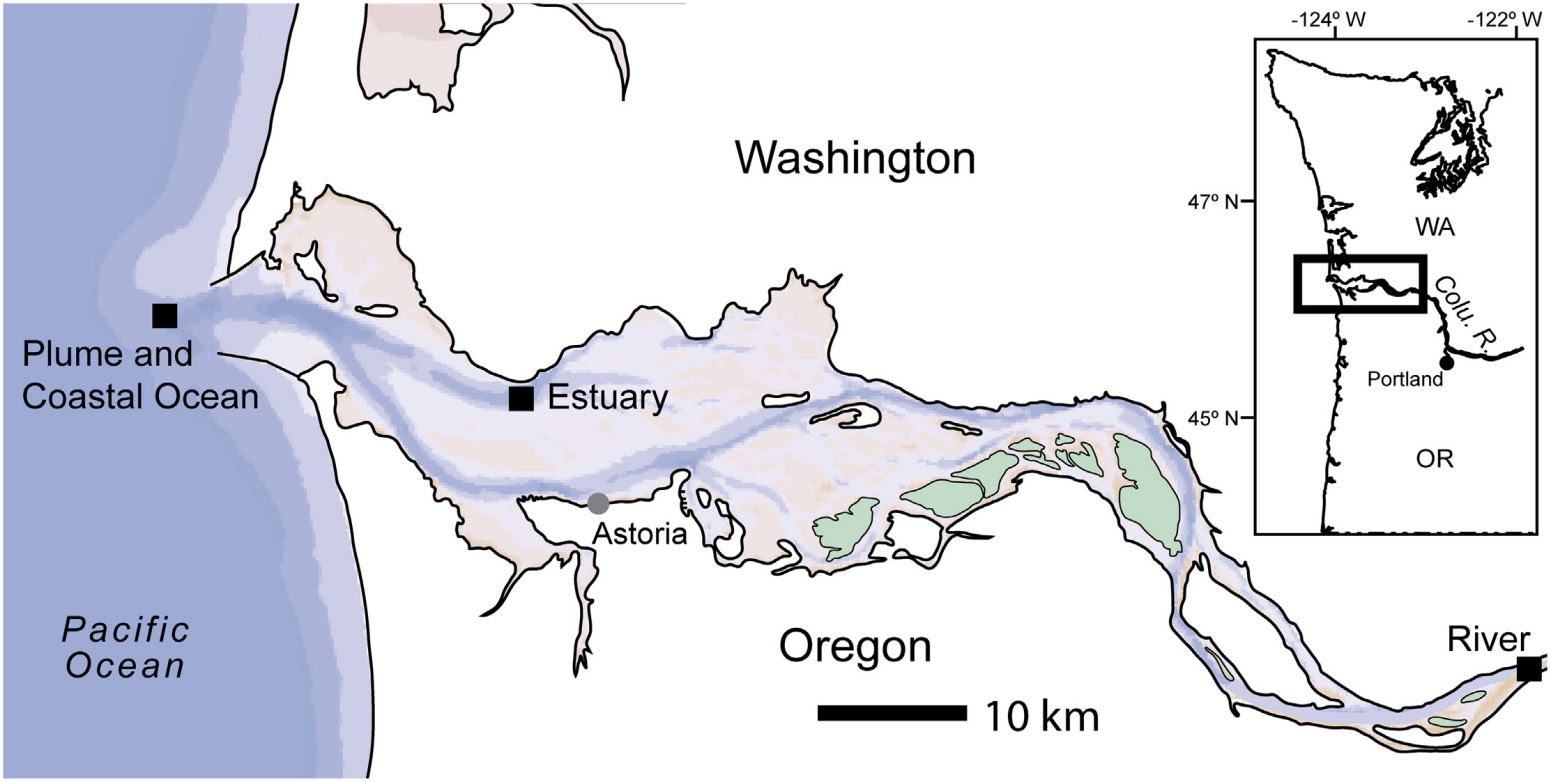
Map of sample locations across the Columbia River coastal margin. Sampling sites are denoted by the black squares. The new plume, old plume, and coastal ocean samples were collected from the same location. Samples were collected aboard the R/V *Wecoma* between August 1^st^ and 8^th^, 2010.

Five samples for metagenomic and metatranscriptomic analyses were collected from the Columbia River, estuary, and plume (latitude: 46.134° to 46.239°, longitude -123.322° to -124.161°, Fig 1) as part of the NSF-funded Science and Technology Center for Coastal Margin Observation and Prediction (CMOP). Samples were collected aboard the R/V *Wecoma* between August 1^st^ and 8^th^, 2010. Water was collected using a high volume low-pressure pump with an attached Seabird 911^+^ conductivity-temperature-depth (CTD) sensor. With each CTD cast, depth profiles of salinity, temperature, turbidity, dissolved oxygen (DO), and fluorescence were recorded. Surface samples (0.5 m) were collected in the freshwater portion of the river and within the north channel of the estuary, For the plume, samples were collected on ebb tides from a fixed location off the coast (latitude 46.239°, longitude -124.161°). Surface waters with salinity less then 31 are considered inside the plume [18]. Salinity was monitored and surface samples were taken at a salinity of 15 to represent a new plume and at 25 to represent an older plume that has a higher fraction of ocean water. A sample was also taken below plume waters (16 m) at a salinity of 33 using a pump from a towed underwater vehicle. Water was collected in carboys and immediately pre-filtered through a 142 mm diameter, 3.0 μm pore size polycarbonate filter (Sterlitech) and then collected on a 142 mm diameter, 0.2 μm pore size polycarbonate filter (Sterlitech). Filters were folded and placed in 15 ml tubes with approximately 10 ml of RNAlater, and stored at -80°C until extraction. Approximately 2.5 to 5 l of water was filtered for each sample and samples were filtered and preserved within 30 minutes of collection to ensure preservation of mRNA. For extraction, the 0.2 μm filters were thawed and rinsed with sterile phosphate buffered saline or water, depending on salinity of the sample, to remove RNAlater. Cells that became dislodged after rinsing were captured on a 0.2 μm Sterivex-GP (Millipore) filter and extracted with the polycarbonate filter. One quarter of the polycarbonate filter was used to extract DNA for a metagenome and the remaining was used for RNA extraction. Also, 1 to 6 l of additional sample water was filtered through 0.2 μm Sterivex-GP (Millipore) filters for DNA to be used in 16S rRNA amplicon pyrosequencing. These filters were extracted, amplified using 16S rRNA gene specific primers, pyrosequenced, and analyzed as described in Fortunato et al. [7] and Fortunato et al. [8]. In addition, water was collected and analyzed for a suite of environmental variables including chlorophyll *a*, total dissolved nitrogen (TDN) and phosphorus (TDP), dissolved organic carbon (DOC), particulate organic carbon (POC) and nitrogen (PN), and bacterial production rate as described previously [8].

RNA was extracted using a modified protocol from the RNeasy kit (Qiagen) as described in Poretsky et al. [21]. Briefly, the thawed, rinsed, 0.2 μm filter was cut into pieces using a sterile blade and placed in a 50 ml tube with RLT buffer (Qiagen) and RNA beads (MoBio). The tube was vortexed for 10 min and centrifuged twice at 5000 rpm. The lysate was placed in a new tube with one-volume 100% ethanol and pushed through a 20-gauge needle several times to shear the RNA. The extraction then followed the RNeasy kit (Qiagen) according to manufacturer instructions. DNA was removed from the extraction using a Turbo-DNase kit (Ambion). A final volume of 100 μl was extracted. Ribosomal RNA (rRNA) was removed from RNA samples using subtractive hybridization as described in Stewart et al. [22]. Sample-specific rRNA probes were constructed from DNA collected on 0.2 μm Sterivex filters. After rRNA removal, RNA concentration was determined on an Agilent 2100 Bioanalyzer. DNA was extracted using a phenol-chloroform extraction method adapted from Zhou et al. [23] and Crump et al [24]. DNA and RNA aliquots (~1 μg) were then sent to Integrated Genomic Services (IGS) at the University of Maryland for sequencing on an Illumina HiSeq 1000 system. Resulting libraries were paired-end, non-overlapping. The average single read length was 100 basepairs (bp).

Metagenomic and metatranscriptomic libraries were assembled using CLC Genomics Workbench (v 4.9) with a minimum length fraction of 80% and a similarity of at least 95%. The minimum contig length was set to 250 bp. Assembled contigs from each library were submitted to the DOE Joint Genome Institute’s Integrated Microbial Genome Metagenomic Expert Review (IMG/MER) annotation pipeline for Open Reading Frame (ORF) identification and functional and taxonomic annotation [25]. Sequences from each library were mapped to ORFs using Bowtie2 v. 2.0.0-beta5 [26] using an end-to-end alignment and default settings to determine the number of sequences per annotated ORF. Prior to mapping, duplicate reads with 100% nucleotide identity and exact length were removed using the program FastUniq v. 1.1 [27].

To identify rRNA in metatranscriptomes, reads were mapped to SILVA SSU and LSU databases (Release 108) [28] using Bowtie2 [26] with a local alignment and default settings. Identified rRNA reads were removed from each metatranscriptome using custom Perl scripts. Ribosomal RNA reads were identified in metagenomes but not removed. Once metagenomic rRNA reads were identified, we characterized them by mapping to the Greengenes 2011 taxonomic database [29] and a manually curated freshwater taxonomic database (K.D. McMahon pers. comm.) [30] using Bowtie2. 16S rRNA sequences were then taxonomically identified with MOTHUR v. 1.21 [31] using the above mentioned Greengenes and freshwater taxonomic databases. Taxonomic assignments with less then 80% confidence were marked as unknown.

ORFs from each sample were annotated via BLAST to the Clusters of Orthologous Groups (COG) database [32] and to the KEGG ontology (KO). Annotations with minimum requirements of an e-score of 1e^-10^, 30% amino acid identity, and alignment length of 40 amino acids were included in functional analyses. To account for differences in read abundance between samples, COG abundances in each metagenomic library were normalized as described by Eiler et al. [12] by dividing the number of hits to each COG by the average number of hits to 35 single-copy COGs (S1 Table) for each library. Metatranscriptomic libraries were normalized using the following ratio: (number of hits to each COG/total annotated transcripts)/(average number of hits to 35 COGs in metagenome/total annotated metagenomic reads). KO abundances were normalized by dividing each KO hit by the number of hits to DNA-directed RNA polymerase, beta subunit gene (*rpoB*). For metatranscriptome, KO abundances were normalized as described for COGs except using the number of hits to *rpoB*. Bray Curtis similarities were calculated based on the normalized abundance of COG functions and were used for hierarchical clustering (complete-linkage method). Similarity calculations and clustering were done using the statistical program R (v. 3.2.1) [33] with the R package *vegan*. Regression analysis was also carried out using R (v. 3.2.1)

Raw metagenomic and metatranscriptomic read data is available through the European Nucleotide Archive (ENA) under sample accession numbers ERS709853 through ERS709862. Assembled and annotated contigs are publically available via IMG under GOLD study ID Gs0084963.

## Results

To determine the metabolic potential and gene expression patterns of microbial communities across the salinity gradient of the Columbia River coastal margin, we collected five water samples from 0 to 33 salinity from river to ocean and generated both metagenomic and metranscriptomic libraries (Fig 1). For the two plume samples, new plume refers to the lower salinity sample (15.4), while old plume refers to the higher salinity sample (25.4), where more mixing with ocean water occurred. Across the salinity gradient, there were some differences in other environmental parameters (Table 1). Although there was some variability, both TDP and TDN generally increased from river to ocean. The highest POC/N concentrations were observed in the river and estuary, with river POC nearly twice as high as the plume and coastal ocean. Bacterial production was highest in the coastal ocean sample, and was over four times higher than in the other four samples (Table 1).

**Table 1:** Sample environmental data and sequencing statistics.

	River	Estuary	New Plume	Old Plume	Coastal Ocean
Date Collected	8/4/10	8/1/10	8/8/10	8/7/10	8/7/10
Salinity	0.06	3.9	15.4	25.4	33.3
Temperature (°C)	20.7	19.4	15.5	13.4	8.5*
Depth (m)	0.5	0.5	0.5	0.5	16
DO (mg/L)	5.91	4.95	3.94	5.65	7.7*
Chlorophyll a (μg/L)	10.34	3.73	3.03	6.31	3.73
TDP (μM)	0.20	0.50	1.15	0.74	2.29
TDN (μM)	13.20	12.70	18.80	11.90	34.80
POC (μg/L)	748.0	566.9	353.2	402.3	405.3
PN (μg/L)	314.4	205.1	95.0	125.5	132.1
DOC (mg/L)	1.65	1.57	1.39	1.41	1.29
Bacterial Production μg/L/hr	0.63	0.48	0.78	0.44	4.73
*Total Paired QC Reads (10* ^*6*^ *)*					
DNA	60.1	59.9	84.1	65.7	60.7
cDNA	12.7	10.9	20.5	11.8	6.5
*Percent rRNA*					
DNA	0.5	2.0	0.4	1.0	1.3
cDNA	85.2	87.5	69.8	81.3	80.8
*Reads annotated to COGs (10* ^*6*^ *)*					
DNA	9.2	9.3	17.3	15.1	13.8
cDNA	0.2	1.7	0.7	0.8	0.2

Environmental parameter abbreviations are as follows: DO, dissolved oxygen; TDP, total dissolved phosphorus; TDN, total dissolved nitrogen; POC, particulate organic carbon; PN, particulate nitrogen; DOC, dissolved organic carbon. The total number of annotated reads for each sample includes both paired and single reads.

* Cast data was unavailable, parameters used are from a CTD cast taken on a different day from a similar depth and location.

Illumina sequencing of metagenomic samples resulted in an average of 66.3 million paired-end reads per library. The metatranscriptomic samples averaged 67.7 million paired-end reads, but this was reduced to 12.5 million after removal of ribosomal RNA sequences, which on average made up 80.9% of the total sequences in each library (Table 1). Reads were assembled into contigs with the resulting number of contigs ranging from 502,915 to 722,195 for the metagenomes and 9722 to 91,349 for the metatranscriptomes. Open reading frames (ORFs) were determined from the contigs and were annotated against the COG and KO databases. Dereplicated reads for each library were then mapped back to the ORFs to determine read abundance. The number of reads annotated against the COG database averaged 12.9 million for the metagenomes and 725,000 for the metatranscriptomes. To account for differences in the number of annotated reads between samples, read abundances were normalized against the average number of hits to 35 COGs for single copy housekeeping genes as described above. Normalized COG abundances were then used to compare taxonomic and functional profiles across samples. To look more closely at individual metabolic processes, we used KO annotations to determine the abundance of specific genes across the salinity gradient.

**Fig 2:**
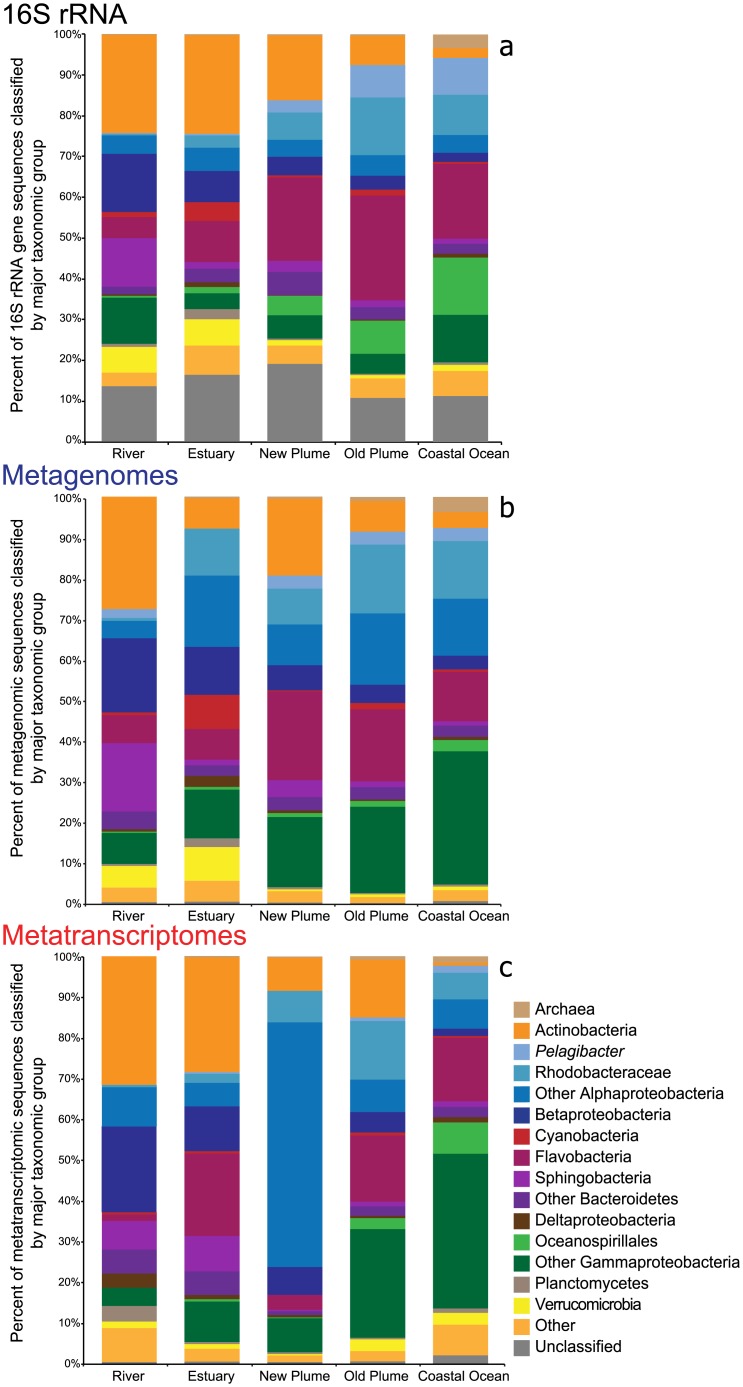
Taxonomic changes across the salinity gradient. Bar charts represent the taxonomic changes seen in a) 16S sequences identified from metagenomes, b) COG annotated sequences from metagenomes, and c) COG annotated sequences from metatranscriptomes.

Taxonomic profiles from the 16S sequences identified in the metagenomes showed a dramatic change in microbial community composition from river to ocean. Actinobacteria and Betaproteobacteria decreased across the salinity gradient, while Gammaproteobacteria, especially the Oceanospirillales family, increased from river to ocean (Fig 2a). The taxonomic classifications of the COG annotated reads from the metagenomes depicted a similar community composition across the salinity gradient, with only slight differences (Fig 2b). With the contig classifications we saw more Alphaproteobacteria in the lower salinity environments of the estuary and new plume. Additionally, there were fewer annotated reads classified as Bacteroidetes and more as Gammaproteobacteria in the higher salinity samples. Examination of the annotated metatranscriptomes showed slight differences in taxonomy compared to the metagenomes (Fig 2c). This was especially true in the estuary, where there was a higher percentage of Actinobacteria in the metatranscriptome compared to the metagenome. Although Cyanobacteria were present in the estuary metagenome, there was little expression of this group in the metatranscriptome. The high expression of Alphaproteobacteria in the new plume was attributed to one contig. A BLASTX of this contig gave the top hit as being from a SAR116 bacterium, an abundant group in the coastal ocean (Fig 2c). In the coastal ocean metatranscriptome, Gammaproteobacteria were most abundant with little expression of Alphaproteobacteria compared to the plume samples.

**Fig 3:**
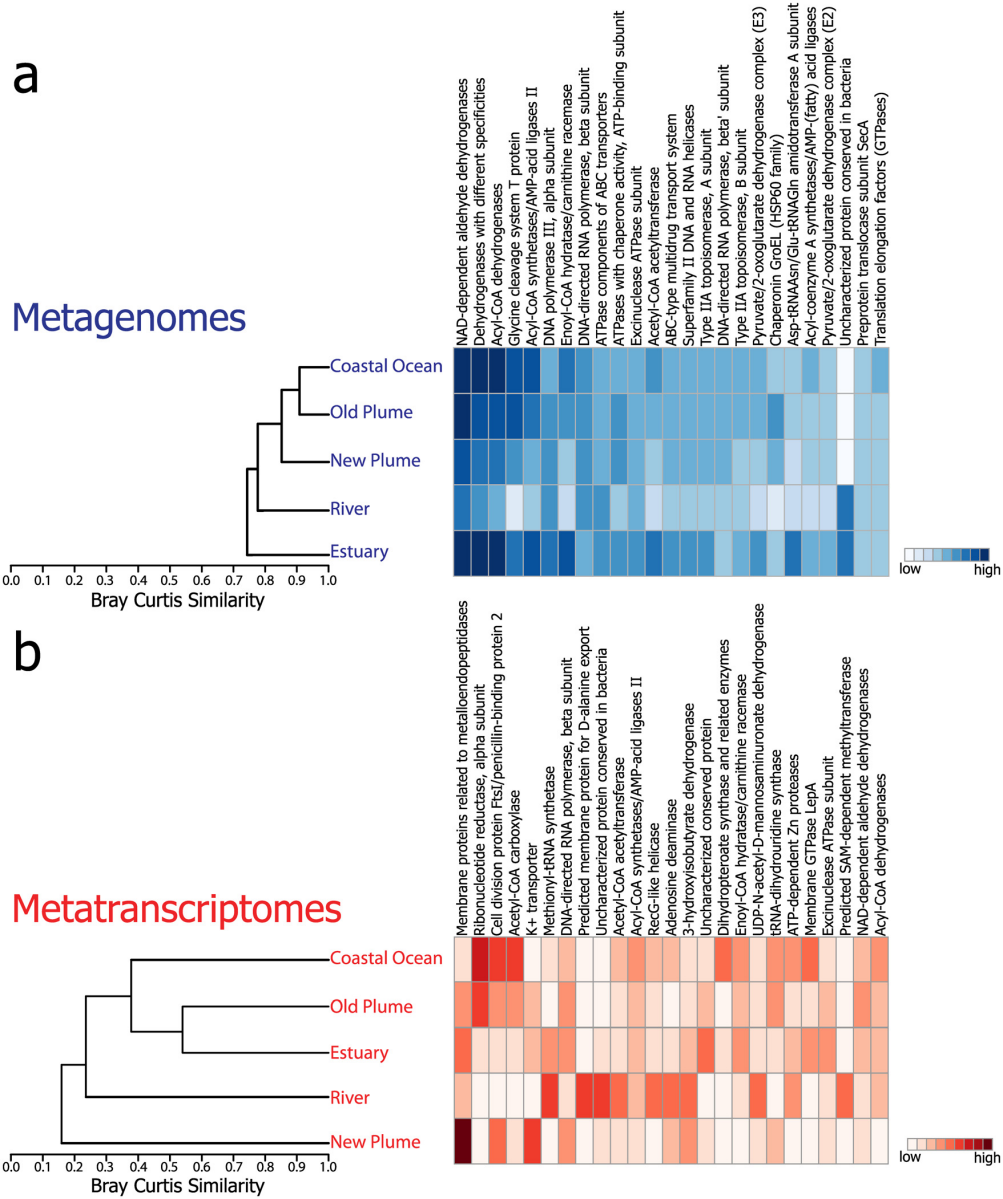
Hierarchical clustering of samples across salinity. Dendrogram and heatmap of a) metagenomes and b) metatranscriptomes based on the normalized abundance of COG functions. Heatmaps depict the top twenty-five most abundant COGs.

Overall across the salinity gradient, the metagenomes were highly similar with an average Bray-Curtis similarity of 82% based on the normalized abundance of COG functions. Metatranscriptomes, however, were less similar with an average of only 31% similarity. Although the metagenomes were highly similar, they were not identical, and hierarchical clustering of the five metagenomes revealed that samples partitioned according to salinity, with the estuary and river metagenomes separating from the higher salinity samples (Fig 3a). The dendrogram based on 16S amplicon data also showed the separation of samples by salinity, with the river and estuary clustering together and away from the plume and coastal ocean (S1 Fig), although the clustering pattern was not exactly the same as the dendrogram based on COG abundance. For the metatranscriptomes, the new plume was the most dissimilar, likely due to the high expression of COG739, which is associated with membrane proteins related to metalloendopeptidases. The river was the next to separate, and the estuary and old plume clustered together (Fig 3b). The corresponding heatmaps of the dendrograms display the twenty-five most abundant COG functions across all samples (Fig 3). For the metagenome samples, the top COGs include those related to energy production and conversion, lipid and amino acid transport and metabolism, transcription, and translation. Abundance patterns reflect overall similarity scores with many COGs having similar abundances across the salinity gradient (Fig 3a). This was not the case for the metatranscriptomes in which there were a few dominant COGs in each of the five samples (Fig 3b). For example the new plume was dominated by COG739, which comprised over 57% of the annotated sequences in the metatranscriptome (Fig 3b). A COG associated with a K^+^ transporter was dominant in the river, while COGs associated with cell membrane biogenesis and lipid transport and metabolism dominated in the coastal ocean (Fig 3b). Overall, the most abundant COGs in the metatranscriptomes were related to cell membrane biogenesis and lipid transport and metabolism, which differ from the more diverse categories seen in the metagenomes (Fig 3).

**Fig 4:**
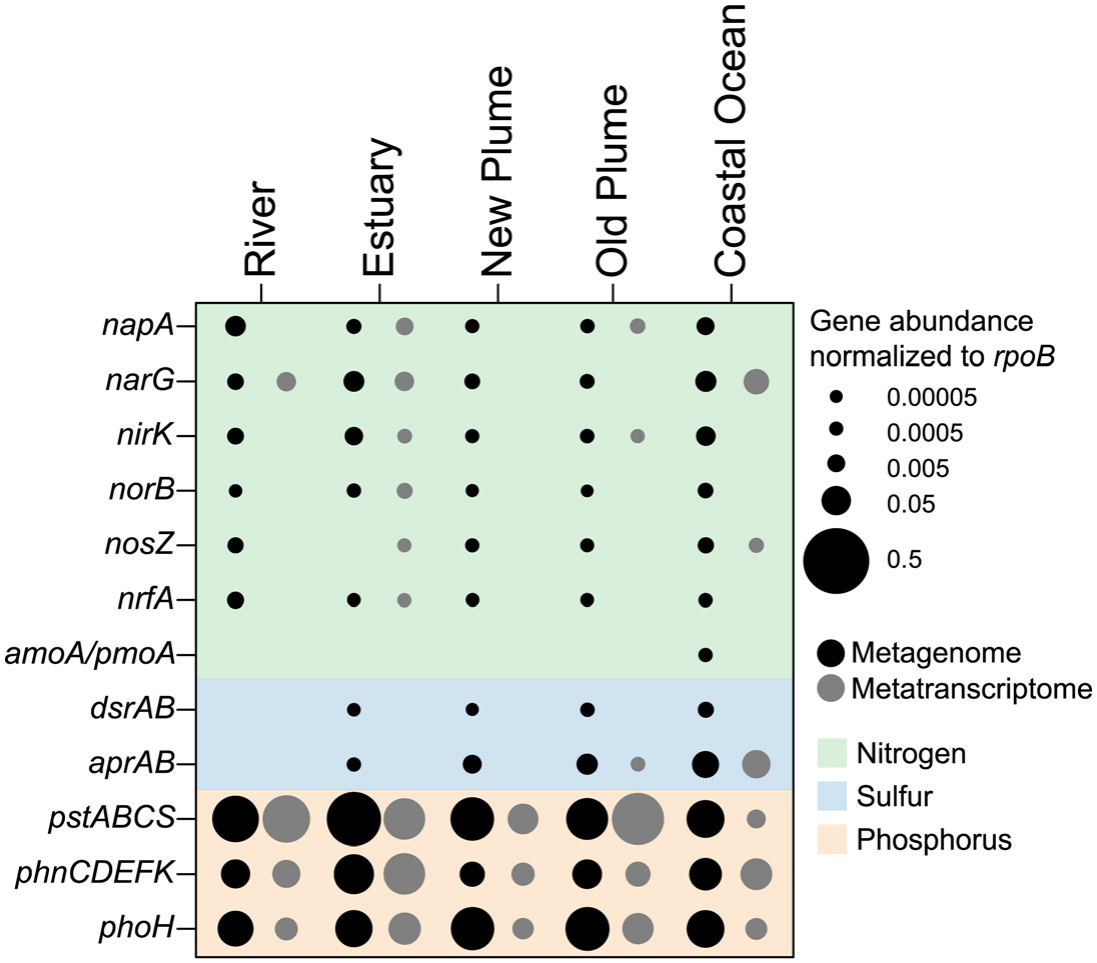
Abundance and expression of key genes involved in nitrogen, sulfur, and phosphorus transport and metabolism. The size of the bubble corresponds to the normalized abundance or expression of each gene. Black bubbles represent metagenomes, gray bubbles represent metatranscriptomes. Gene abbreviations are as follows: *napA* and *narG*, nitrate reductase; *nirK*, nitrite reductase; *norB*, nitric oxide reductase; *nosZ*, nitrous oxide reductase; *nrfA*, nitrite reductase to ammonia; *amoA/pmoA*, ammonia/methane monooxygenase; *dsr*, dissimilatory sulfite reductase; *apr*, adenosine-5′-phosphosulfate reductase; *pst*, high-affinity phosphate transporter; *phn*, phosphonate transport; *phoH*, phosphate starvation-inducible protein.

An assessment of the abundance of nitrogen, sulfur, and phosphorus metabolism and transport genes showed little pattern across the salinity gradient (Fig 4). Nitrogen cycle genes involved in nitrate reduction, denitrification, and dissimilatory nitrate reduction to ammonium (DNRA) were present at some level of abundance in all metagenomes from river to ocean, with no discernable pattern related to changes in salinity (Fig 4). Expression of denitrification genes was observed mostly in the estuary, with the exception of a few genes being expressed in the river, old plume, and coastal ocean (Fig 4). Although our data focused on the community members less then 3μm, smaller particles may persist in the estuary and may be environments for denitrifying organisms bearing the denitrification genes detected in our free-living community. The abundance of sulfur metabolism genes, specifically those for sulfur oxidation (*aprAB*) and sulfur reduction (*dsrAB*) did increase with salinity, with the expression of sulfur oxidation genes in the old plume and even higher expression in the coastal ocean (Fig 4). The abundance of phosphorus transport genes showed no clear pattern, as genes for phosphate and phosphonate transport were both highly abundant and expressed in all samples (Fig 4).

**Fig 5:**
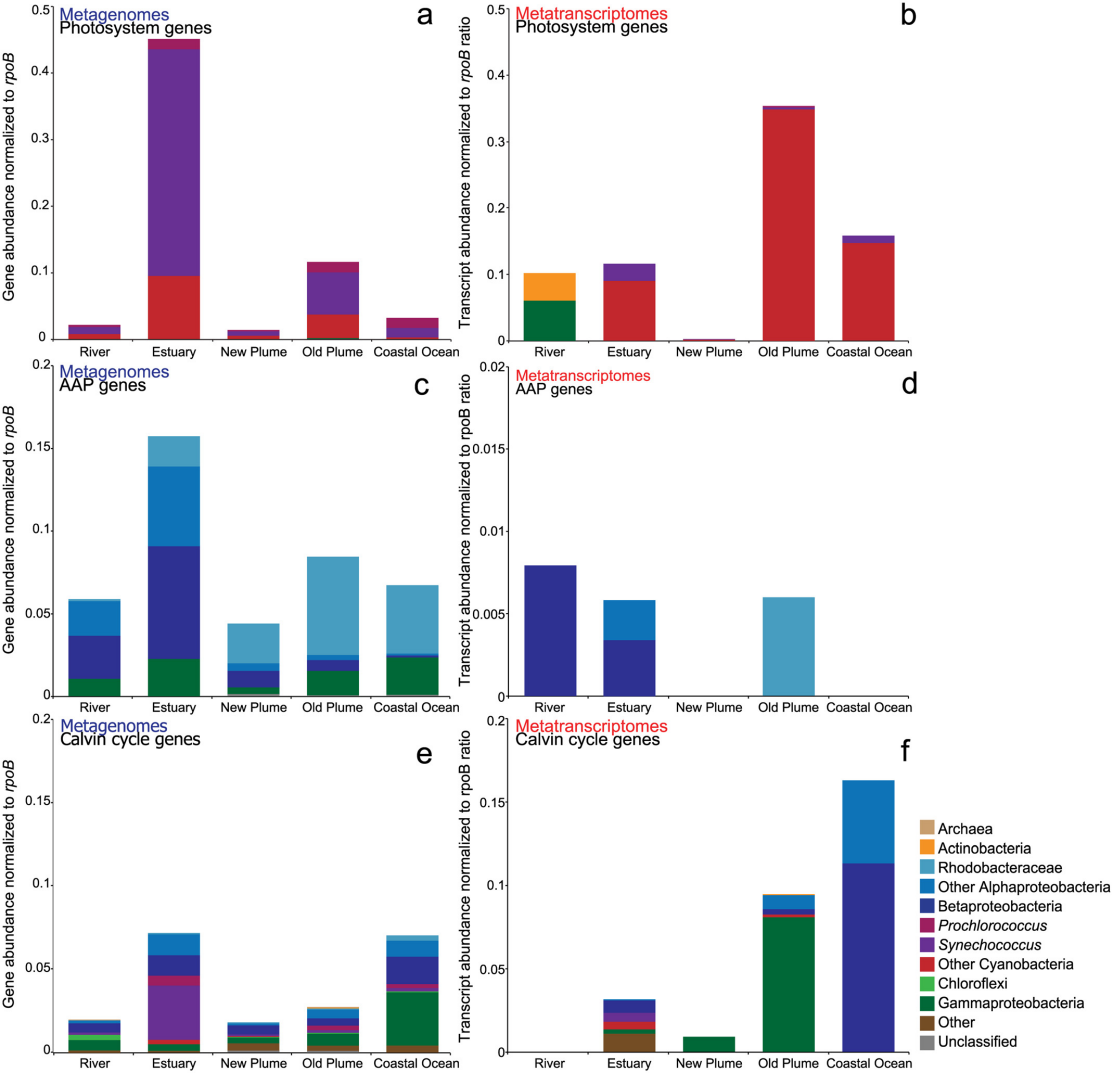
Bacterial photosynthesis and carbon fixation gene abundance and expression. Abundance (a) and expression (b) of oxygenic photosynthesis genes, represented by photosystem genes *psa* and *psb*. Abundance (c) and expression (d) of aerobic anoxygenic photosynthesis (AAP) genes, represented by *pufABLM*, and *puhA* genes. Abundance (e) and expression (f) of carbon fixation via the Calvin Benson Bassham cycle, represented by *rbcSL* and *prk* genes.

Some of the few functional differences between metagenomes were observed in the abundance of photosynthesis genes. We observed that the abundance and also the expression of genes for bacterial photosynthesis (oxygenic and anoxygenic) and carbon fixation varied across samples (Fig 5). The highest abundance of oxygenic photosynthesis genes, photosystems I and II, was observed in the estuary and was mostly attributed to *Synechococcus*. This corresponds well to the abundance of Cyanobacteria in the estuary metagenome (Fig 2a and 2b). Expression of photosystem genes, however, was highest in the old plume and coastal ocean. These transcripts could not be attributed to a specific genus and thus were classified with other cyanobacteria (Fig 5a and 5b). Indicator genes (*pufABLM*, *puhA*) for anaerobic anoxygenic phototrophs (AAPs) were also present across the salt gradient, and were highest in the estuary (Fig 5c). Expression of these genes was low and was detected only in the river, estuary, and old plume samples (Fig 5d). Taxonomically, there was a change in AAP bacteria from Betaproteobacteria in the river to Alphaproteobacteria in the higher salinity metagenomes, with Gammaproteobacteria evenly distributed. This pattern was also observed in the metatranscriptomes, with AAP genes classified as those of Betaproteobacteria in the river, of both Alpha and Betaproteobacteria in the estuary, and just Alphaproteobacteria (family Rhodobacteraceae) in the old plume (Fig 5c and 5d). Genes functioning in carbon fixation via the Calvin cycle were most abundant in the estuary and coastal ocean (Fig 5e). Expression of these genes was highest in the old plume and coastal ocean (Fig 5f). Taxonomically, Calvin cycle genes were classified to Cyanobacteria as well as to Alpha, Beta, and Gammaproteobacteria across samples (Fig 5e and 5f).

**Fig 6:**
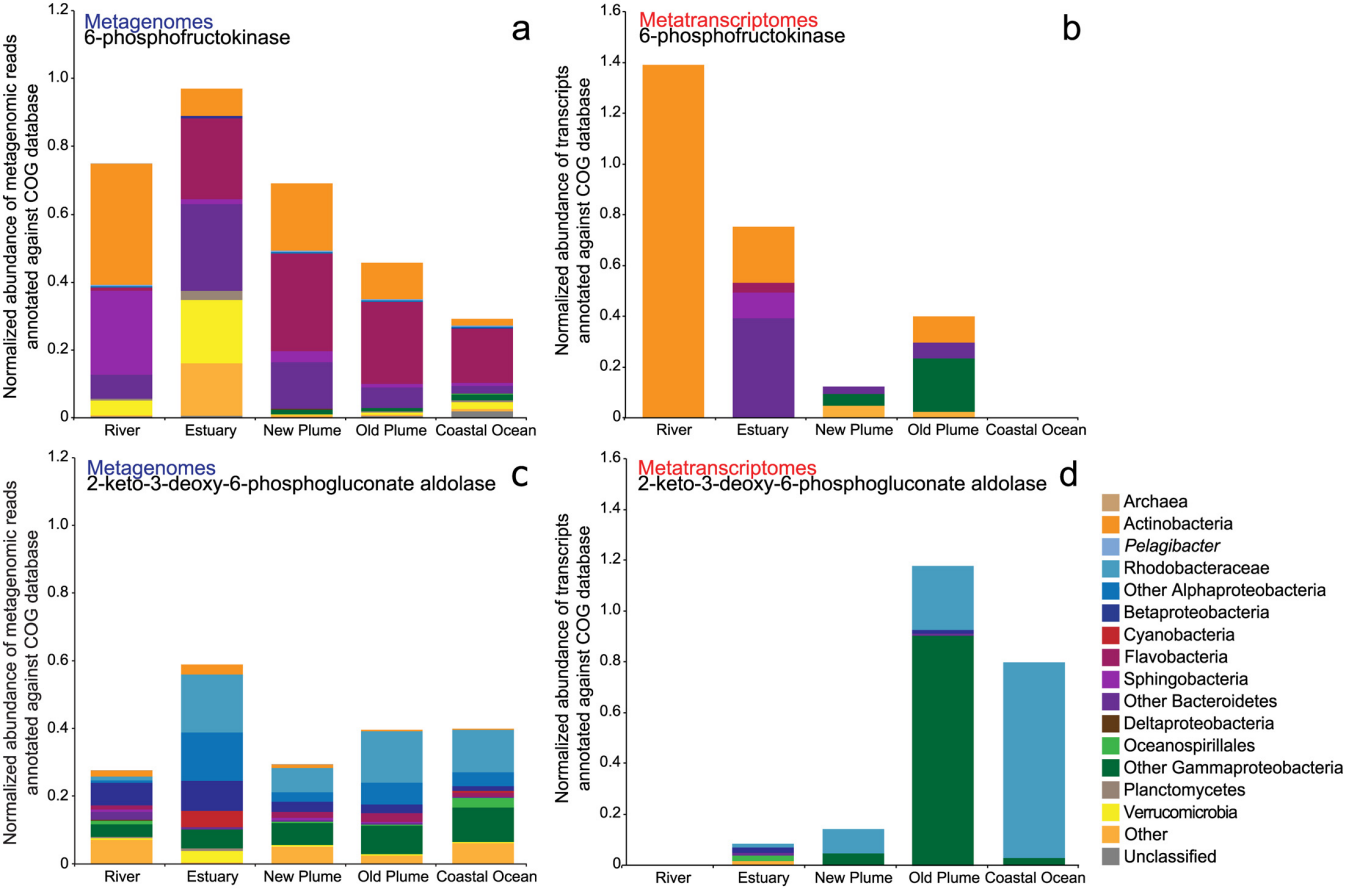
Gene abundance and expression for key indicator enzymes associated with glycolysis. Abundance (a) and expression (b) of the key enzyme in the Embden-Meyerhof (EMP) pathway, 6-phosphofructokinase, represented by COG205. Abundance (c) and expression (d) of the key enzyme in the pathway Entner-Doudoroff (ED) pathway, 2-keto-3-deoxy-6-phosphogluconate aldolase, represented by COG800.

Although the metagenomes of this study were highly similar from river to coastal ocean, there were differences in the abundance of a small number of central metabolism, transport, and osmoregulation genes. Recent metagenomic studies [11, 12] uncovered genomic differences between freshwater and marine bacteria. We looked for these differences in our metagenomes and sought to determine if they were reflected in gene expression patterns. Glycolysis is an important step in bacterial central metabolism and bacteria utilize different pathways for carrying out this process. The two most common are the Embden-Meyerhof (EMP) and Entner-Doudoroff (ED) pathways. For the EMP pathway, the gene abundance of the key enzyme, 6-phosphofructokinase (COG0205), appeared to decrease with salinity, with the highest abundance in the river and estuary and lowest in the coastal ocean (Fig 6a). A regression of the abundance of COG0205 with salinity gave an *R* ^*2*^ of 0.83 (*p* = 0.03). A similar trend with salinity was observed for gene expression of COG0205, although this trend was not significant (*R* ^*2*^ = 0.71, *p* = 0.07, Fig 6b). The relationship with salinity was not as strong for the abundance of 2-keto-3-deoxy-6-phosphogluconate aldolase (COG0800), the key enzyme for the ED pathway. Gene abundance for COG0800 was lowest in the river, but the trend with salinity was not significant (*R* ^*2*^ = 0.01, *p* = 0.94, Fig 6c). Conversely, gene expression did show a pattern, albeit not significant, with salinity, with COG0800 highly expressed in the old plume and coastal ocean (*R* ^*2*^ = 0.71, *p* = 0.07, Fig 6d). This switch between the EMP and ED pathways is consistent with the taxonomic change across the salinity gradient. The EMP pathway enzyme is more prevalent in Actinobacteria and Bacteroidetes, which were more abundant in lower salinity waters. The ED pathway is more common in Alphaproteobacteria and Gammaproteobacteria, which increased in abundance towards the coastal ocean (Fig 6) [34].

**Fig 7:**
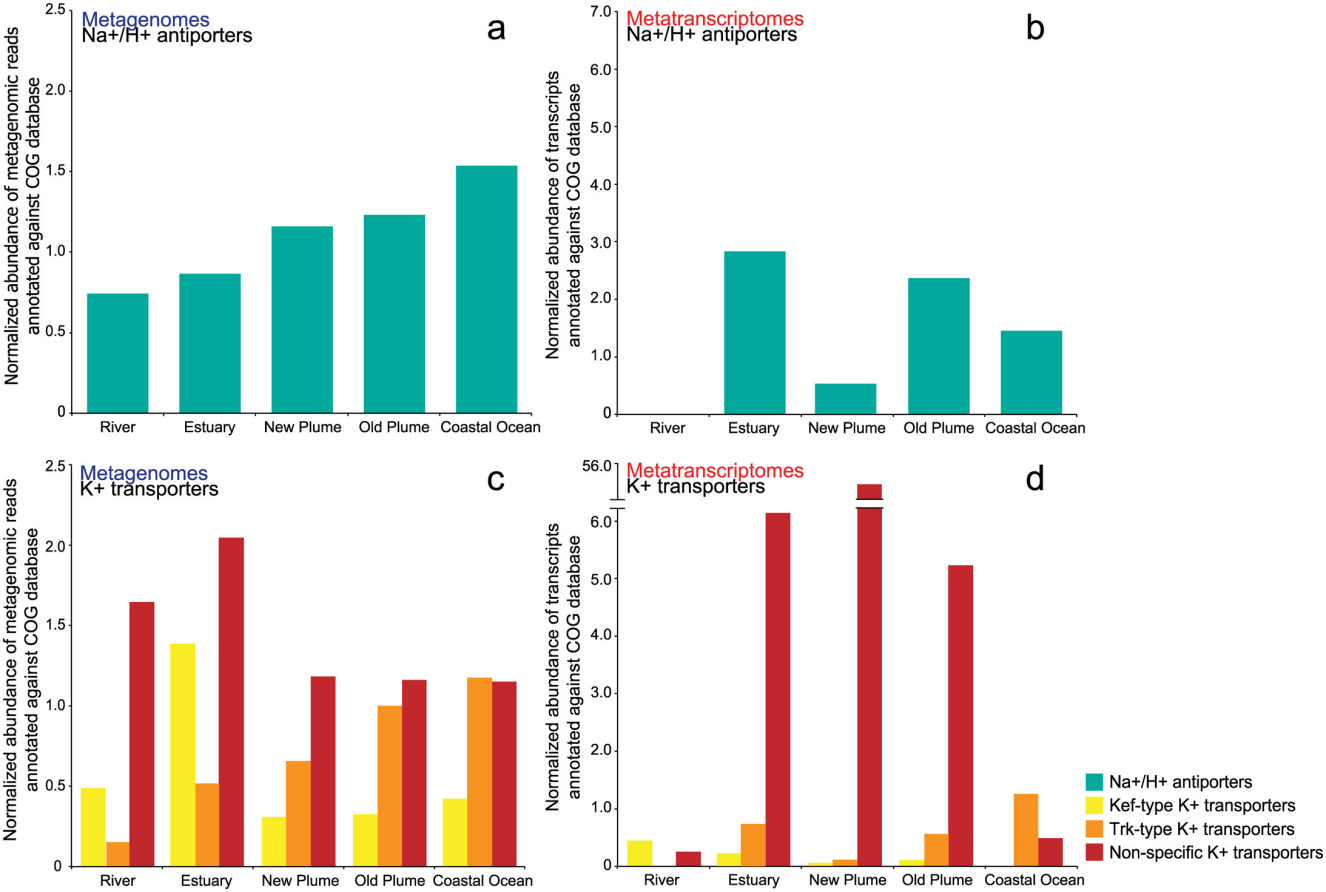
Changes in osmoregulators with salinity. Abundance (a) and expression (b) of Na^+^/H^+^ antiporters, represented by COGs: 2111, 1006, 1863, 2212, 1320, 3004, 3067, 1055, 1757, and 1563. Abundance (c) and expression (d) of K^+^ transporters, represented by COGs: 569, 3158, 2060, 2156, 2216, and 168.

Abundance and expression of some transporter genes also changed over the salinity gradient. The abundance of sodium transporters steadily increased with salinity from river to coastal ocean (*R* ^*2*^ = 0.96, *p* < 0.01, Fig 7a). Expression of these genes was variable and showed no trend with salinity. Expression was highest in the estuary, old plume, and coastal ocean (Fig 7b). Potassium transporter gene abundance showed no pattern from river to ocean. A regression of total potassium transporter abundance with salinity showed no significant trend (*R* ^*2*^ = 0.06, *p* = 0.7, Fig 7c). Expression of potassium transporter genes, however, was highest in the estuary and plume, suggesting active osmoregulation by freshwater microbes as they are exposed to higher salinity waters (Fig 7d). Additionally, the abundance of transporter genes for glycine betaine and proline, key osmoprotectants for bacteria, showed a significant relationship with salinity (*R* ^*2*^ = 0.99, *p* < 0.01).

The abundance and expression of some metal transport genes also varied among samples. Fe^+2^ transporter genes were most abundant in river and estuary and less abundant in the higher salinity samples. The abundance of Fe^+3^ transporter genes was lowest in the river and highest in the estuary (S2 Fig). Expression of these genes was variable with salinity, however Fe^+2^ transporter gene expression was highest in the river and lowest in the coastal ocean while Fe^+3^ transporter gene expression was highest in the coastal ocean and lowest in the river (S2 Fig). Gene abundances for Co, Mg and Ni transporters did not change drastically across the salinity gradient, while Zn and Mn transporters were lowest in the river and highest in the old plume (S2 Fig). Expression of these transport genes was highly variable with no visible relationship with salinity. In addition, recent papers show the metabolic importance of manganese to estuarine and marine microbial communities [35, 36]. Specific manganese transport genes *mntH* and *sitABCD* were observed across the salinity gradient, with a change from *mntH* in lower salinity waters to the *sitABCD* transporter complex in the plume and coastal ocean (S3 Fig). Expression was only observed for the *mntH* gene and was highest in the estuary and new plume (S3 Fig).

## Discussion

For microbial communities, salinity presents a significant barrier for dispersal of taxa from fresh to marine waters. Many studies have shown the clear taxonomic transition that occurs across a salinity gradient [1, 8, 11, 37, 38], but the functional differences between these communities and the reasons behind these differences have not been fully explored. Recent studies comparing metagenomes from freshwater lakes and marine environments and studies of metagenomes across the Baltic Sea salinity gradient showed functional differences in core metabolism and respiration genes as well as genes involved in nutrient uptake and processing [11, 12]. Although we detected a few of the same functional differences among metagenomic samples, our results suggest that free-living microbial communities of this river-dominated coastal margin environment were functionally very similar from river to ocean based on genomic potential (Fig 3a, Bray Curtis similarity of 74–91% based on normalized abundance of COG functions). However, the corresponding metatranscriptomic data showed highly variable patterns of gene expression, which were dominated by the expression of a few key genes at each salinity. This study is the first to analyze both the functional diversity and gene expression patterns of free-living microbial communities across changing salinities. Taken together, our results indicate taxonomically distinct yet functionally similar communities from river to ocean, with variable gene expression depending on the environmental conditions.

Estuaries and plumes represent transitional environments for freshwater and coastal ocean microbial communities. Microbial communities within these transition zones are exposed to gradients in salinity, temperature, and nutrients as well as ever-changing physical conditions unique to each habitat. The Columbia River coastal margin is a fast moving, river-dominated system with high river flow and short estuary residence times. Thus, the high functional similarity of the free-living microbial communities (Fig 3a), especially at the intermediate salinities of the estuary and plume can be attributed to these fast changing environmental conditions. At the time of sampling in August 2010, average river flow was 3910 m^3^s^-1^ and the estuarine residence time was 8.2 days. Although this residence time may be on the same scale as typical doubling times of free-living populations (average 9.1 d) [39], these conditions present microbial communities with little time for local functional adaptation at different salinities as they are mixed through the estuary and into the coastal ocean. Previous studies of 16S profiles across the salinity gradient of the Columbia River coastal margin demonstrated that communities at intermediate salinities are a mixture of river and coastal ocean bacterial end-members [7, 40]. The establishment of an estuarine specific community was only observed in particle attached bacteria, the residence time of which is much longer due to particle entrainment in the estuary turbidity maximum [41]. Hence, the metabolic similarity across the salinity gradient could be explained by the mixing of fresh and coastal ocean free-living communities that share metabolic capabilities required for existence in pelagic environments. Additionally, our sampling of free-living communities across the salinity gradient of the Columbia River coastal margin did not encompass the large metabolic gradients seen in other river systems. For example, Ghai et al [14] compared a freshwater metagenome from the Amazon to metagenomes from the Global Ocean Survey (GOS) and found large differences between river and marine waters, specifically a high abundance of genes associated with microbial heterotrophy in fresh versus marine waters. In the Columbia river, however, the majority of organic matter is not terrestrial material, but derived from river phytoplankton blooms [42], and thus genes for the breakdown of terrestrial-derived organic matter such as cellulose and lignin may not be as prevalent as in the Amazon. All samples were taken in the photic zone and did not span large oxygen, depth, or nutrient gradients where there would be a large change in metabolic function, again leading towards metagenomes displaying very similar functions across salinity. Thus, highly similar functional profiles from river to ocean appear to be driven by a combination of a fast moving river with short estuary residence times and a limited metabolic gradient from river to ocean.

Although functional potential was highly similar, the metagenomes were not completely identical and we found differences in the abundances of a few genes for core metabolic pathways, metal transport, and osmoregulation. Dupont et al. [11] reported a metagenomic study of the Baltic Sea salinity gradient where estuary residence time ranged from 3–30 years, which presumably could allow for greater functional adaptation at different salinities. They showed key differences across salinity in the abundance of genes for bacterial respiration, glycolysis, osmoregulation, and metal transport. Despite the large difference in residence times between the Columbia River estuary (days) and the Baltic Sea (years), some of the same functional differences were observed in this study. These differences, however, made up a small percentage of the annotated metagenomic sequences, and the metagenomes were for the most part highly similar.

One difference we detected in the metagenomes was in genes coding for the biosynthesis of quinone dehydrogenases, which are essential components of the bacterial electron transport chain. As in the Dupont et al. [11] study, our study found that the abundance of genes for Na^+^-translocating (NQR) dehydrogenases increased with salinity (*R* ^*2*^ = 0.86, *p* = 0.02). The abundance of genes for H^+^-translocating (NDH) NADH:quinone dehydrogenases, however, did not significantly decrease with increasing salinity (*R* ^*2*^ = 0.62 *p* = 0.11). Expression of these quinone dehydrogenases also showed no significant relationship with salinity. The metabolism of glucose is an important pathway for energy production in bacteria, but different pathways for glycolysis operate with differing costs and benefits for bacterial populations [34]. Again, similar to the Dupont et al. study, we observed a nearly linear trend with salinity in the abundance of genes for the EMP pathway, decreasing from river to ocean (*R* ^*2*^ = 0.83, *p* = 0.03, Fig 6a). This linear trend with salinity was also observed for the expression of genes for both the EMP and ED pathways, but was not significant (*R* ^*2*^ for both = 0.71, Fig 6c and 6d). Taxonomically, EMP genes and transcripts were from Actinobacteria and Bacteroidetes while those encoding ED enzymes were from Gammaproteobacteria (Fig 6). This taxonomic distinction was also found in a study of glycolysis pathways of sequenced genomes, in which the more prevalent EMP pathway genes were found mostly in Actinobacteria, Bacteroidetes, and Firmicutes while ED pathway genes were observed in Proteobacteria [34]. This study also showed that although the ED pathway yields less ATP, it requires synthesis of several-fold fewer proteins to catalyze reactions compared to the EMP pathway and is more common in microbes with alternate forms of ATP production (e.g. photosynthesis, proteorhodopsins) [34], which might explain the prevalence of this pathway in the higher salinity plume and coastal ocean samples. Our study expands on the observations made by Dupont et al. by showing that the differences in gene abundance of these important compounds and pathways are also reflected in gene expression patterns and thus the functional activity of microbial communities across the salinity gradient. In addition, it is interesting that variation in bacterial respiration and core metabolism pathways with salinity were observed in two very different estuaries (Columbia and Baltic) with different residence times and presumably very different selective pressures on microbial communities. These results indicate that phylogeny and metabolic potential are not independent of each other. For example, since Actinobacteria use the EMP pathway for glycolysis and the percentage of Actinobacteria decreases with increasing salinity then it also follows that we see the same pattern in the abundances of genes for the EMP pathway. Thus, the patterns observed for these functional genes may be purely driven by taxonomic differences across the salinity gradient rather then selective pressures on metabolic function.

Dissolved metals are important for many enzymatic processes in microbes. In the new plume, 57% of annotated metatranscriptomic sequences were attributed to the expression of metalloendopeptidases (COG739) from a SAR116 population (Figs 2c and 3b). SAR116 is one of the most abundant heterotrophic clades in both the open and coastal ocean [43] and was first isolated off the Oregon coast [44]. Two representative genomes are available in sequence databases [45, 46], both of which were present and especially abundant in the plume and coastal ocean samples, where they constituted 1–3% of annotated metagenomic sequences. Although expression of genes within this group was highest in the new plume (57% of annotated sequences in the metatranscriptome), SAR116 also made up about 3% of annotated sequences in the old plume metatranscriptome. In addition to expression of metalloendopeptidase genes, genes for zinc, iron, and nickel transporters classified to SAR116 were also expressed in both plume metatranscriptomes, again pointing towards the importance of the Columbia River as a source of dissolved metals to the coastal ocean. Genes classified to SAR116 for amino acid and lipid transport and metabolism were also highly abundant and expressed in the plume. In a study of DOC transformation by coastal bacteria, SAR116 was found to assimilate different individual DOC compounds, including the osmolyte glycine betaine [47]. Genes for glycine betaine/proline transporters classified to SAR116 were detected in both plume metagenomes. The fact that an abundant marine group like SAR116 was most abundant at intermediate salinities (new plume = 15.4, old plume = 25.4, Table 1) and was expressing genes for amino acid, lipid, and metal transport and metabolism shows the importance of river inputs to the coastal ocean [7, 8, 18] and reinforces the view of the plume as a hotspot for primary productivity.

Microbes regulate osmotic pressure through the transport of cations across the cell membrane as well as through the uptake of osmoprotectants like glycine betaine, proline, and DMSP [48]. As microbial populations are flushed from the river to the coastal ocean, and become mixed into the estuary and plume, the regulation of osmotic pressure is extremely important in order to maintain positive cell turgor and thus cell growth [48, 49]. This likely explains the elevated expression of genes for cation transport at intermediate salinities. In freshwater systems, potassium salts are naturally more abundant then sodium salts and thus potassium based transporters are more prevalent in freshwater while sodium based transporters are found in marine communities [12, 13]. In this study the abundance of sodium ion antiporters increased linearly with salinity (*R* ^*2*^ = 0.96, *p* < 0.01, Fig 7a) as did transporters for the osmolytes glycine betaine and proline (*R* ^*2*^ = 0.99, *p* < 0.01). Sodium transporters and osmoprotectant molecules have been observed in a number of marine metagenomic studies and provide insight into how these communities cope with osmotic stress [13, 50, 51]. Abundance of potassium transporters was variable with salinity, with some transporters having higher abundances in low salinity samples and some showing the opposite (Fig 7c and 7d). Expression of potassium transporters was highest in the estuary and plume, especially in the new plume where expression was almost 10 times higher compared to the other samples (Fig 7d). In both the new and old plume much of this expression could be attributed to freshwater populations, with 99% of potassium transporter genes in the new plume and 83% in the old plume taxonomically assigned to Actinobacteria and Betaproteobacteria. In the estuary, the high expression of potassium transporter genes was attributed to these freshwater groups but also to Bacteroidetes populations, perhaps due the greater presence of particles and labile organic matter in the estuary [39]. High expression of potassium osmoregulators in the estuary and plume depicts the changes freshwater populations undergo as they are mixed into the coastal ocean and thus Fig 7d serves as a visual representation of the osmotic stress experienced by freshwater populations across the salinity gradient.

In this study, we focused only on the microbial communities that were less then 3 μm in size, which in previous studies was used to represent the free-living community [39, 41]. However, particles smaller then 3 μm are present and thus our samples are not completely particle-free. Previous metagenomic studies have shown distinct functional differences between particulate and free-living microbial communities in different environments including estuarine, plume, and oxygen-minimum zones [20, 52]. Smith et al. [20] found that the greater than 3 μm size fraction of a metagenome from the Columbia River estuary was enriched, relative to smaller size fractions, in genes for many anaerobic processes including sulfate reduction, methanogenesis, and genes involved in denitrification, specifically dissimilatory nitrate and cytochrome c-containing nitric oxide reductases. Thus estuarine particles provide anaerobic microzones, which microbial populations utilize [20]. Genes for the anaerobic processes of dissimilatory nitrate reduction and denitrification were present across all samples in this study, but had higher abundances in the estuary (Fig 4). Expression of these genes was elevated in the estuary as well, although the dissimilatory nitrate reductase gene *narG* was also highly expressed in the river and coastal ocean (Fig 4). Without the inclusion of larger particles, which are likely locations for anaerobic processes, our results present only a partial picture of metabolic diversity across the salinity gradient, but are still able to show the expression of denitrification genes in the estuary and potential removal of fixed nitrogen from the system, although expression of these genes was relatively low.

The Columbia River plume and adjacent coastal ocean are highly productive environments, in terms of both primary and secondary production [18]. In this study we observed the highest expression of genes for bacterial oxygenic photosynthesis and carbon fixation in both the old plume and coastal ocean samples. Photoheterotrophy via aerobic anoxygenic phototrophs (AAPs) has also been shown to be abundant and important to biogeochemical cycling in marine, estuarine, and freshwater environments [53–55]. Genes for aerobic anoxygenic photosynthesis were present in all samples but were highest in the particle-rich estuary (Fig 5c). Previous studies have shown AAPs to be associated with particles in coastal ecosystems [55, 56], and in the Columbia River coastal margin Smith et al. [20] found that AAPs were more abundant in the particulate fraction compared to free-living. Taxonomically it is interesting to see a gradual change from the freshwater Betaproteobacteria in the river to mostly Alphaproteobacteria in the plume and ocean samples, with the estuary being a mix of both (Fig 5c and 5d). Much of the abundance and expression of genes for aerobic anoxygenic photosynthesis in the plume and coastal ocean were assigned to the Rhodobacterales family. Some *Rosebacter* isolates, which are part of this family, are known AAPs and Alphaproteobacteria strains previously isolated off the Oregon coast were shown to have *puf* operon genes [57]. The highest expression of aerobic anoxygenic photosynthesis genes was actually seen in the river and genes were taxonomically classified as Betaproteobacteria (Fig 5d). Freshwater AAPs have been shown to be taxonomically distinct from marine groups and include members of the Alpha and Betaproteobacteria [53]. Overall, the abundance and expression of aerobic anoxygenic photosynthesis genes across salinity demonstrates the prevalence and importance of this group for carbon and nutrient cycling in the Columbia River coastal margin.

As with all molecular based approaches there are limitations to using metagenomic and metranscriptomic data to describe communities. Due to the high diversity of our samples, assemblies were limited and the percent of annotated sequences from each metagenome or metatranscriptome was low and variable. This low number of annotated sequences limited our understanding of the metabolic potential and to a greater extent the gene expression occurring in the community and may bias results towards certain functions. As with all meta-omics studies, annotations are also constrained by the databases being queried and thus are restricted to known pathways, which hinders analysis and understanding of all metagenomic and metatranscriptomic sequences in a sample. Given these known drawbacks we attempted to minimize biases between samples through normalization and comparison to multiple protein databases (COG and KO). The data presented here, although not complete, depicts how known genetic functions and pathways change, or do not change, across environmental gradients.

Across the salinity gradient of the Columbia River coastal margin, we observed a pronounced change in the taxonomy of the free-living microbial communities. However, despite this difference in taxonomy from river to ocean, functionally these free-living communities were highly similar. We postulate that the combination of a fast moving river with short estuary residence times resulted in little time for local functional adaptation. This, coupled with little variation in other environmental conditions such as oxygen and DOC (Table 1), resulted in highly similar functional profiles from river to ocean for the free-living community. Despite this overall similarity, differences in the abundance of genes for osmoregulation and glycolysis were observed in the metagenomes and the abundance of these genes showed a significant linear relationship with salinity. Conversely, the metatranscriptomes were extremely variable and transcripts showed little to no relationship with salinity. In conclusion, free-living microbial communities across the Columbia River coastal margin were taxonomically different but metabolically similar, with variable patterns in expression.

## Supporting Information

S1 Fig Hierarchical clustering of samples across salinity. Dendrogram is based on the relative abundance of 16S amplicon sequences.
(PDF)

S2 Fig Metal transporter gene abundance and expression. Abundance (a) and expression (b) of iron transporters, represented by COGs: 1840,1178, 614, 609, 1120, 1918, 370, 3470. Abundance (c) and expression (d) of cobalt, magnesium, nickel, zinc, and manganese transporters, represented by COGs: 5266, 310, 1122, 619, 2239, 598, 4536, 4535, 2967, 1121, 1108, and 4531.
(PDF)

S3 Fig Manganese transporter gene abundance and expression. Abundance (a) and expression (b) of two common manganese transporters, *mntH* and *sitABCD* across salinity.
(PDF)

S1 Table Single copy COGs used for metagenome normalization. (XLS)
