# Supplementary material for: Microbial Gene Abundance and Expression Patterns across a River to Ocean Salinity Gradient
Source: PLoS One. 2015 Nov 4;10(11):e0140578. doi: 10.1371/journal.pone.0140578 (PMC4633275; doi:10.1371/journal.pone.0140578)

S1 Fig. Hierarchical clustering of samples across salinity.

Dendrogram is based on the relative abundance of 16S amplicon sequences.

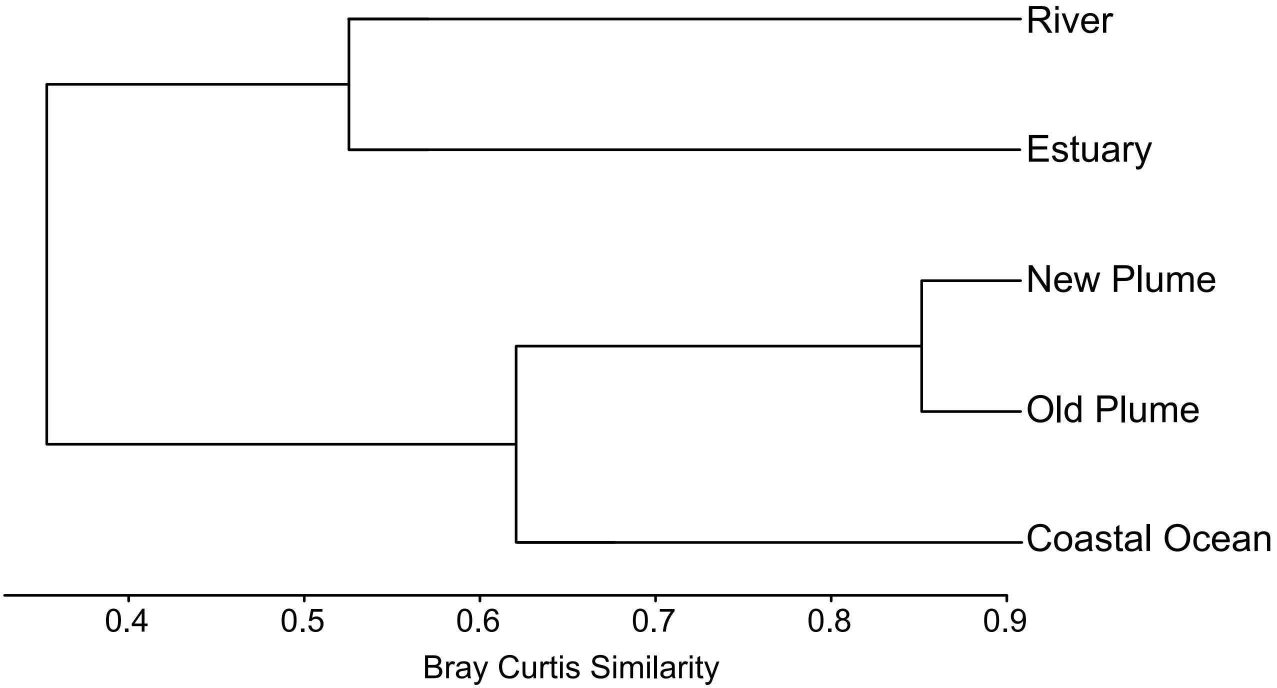

Supplement: S1 Fig — Dendrogram is based on the relative abundance of 16S amplicon sequences. (PDF) [file pone.0140578.s001.pdf]
