# Supplementary material for: Microbial Gene Abundance and Expression Patterns across a River to Ocean Salinity Gradient
Source: PLoS One. 2015 Nov 4;10(11):e0140578. doi: 10.1371/journal.pone.0140578 (PMC4633275; doi:10.1371/journal.pone.0140578)

S2 Fig. Metal transporter gene abundance and expression

Abundance (a) and expression (b) of iron transporters, represented by COGs: 1840,1178, 614, 609, 1120, 1918, 370, 3470. Abundance (c) and expression (d) of cobalt, magnesium, nickel, zinc, and manganese transporters, represented by COGs: 5266, 310, 1122, 619, 2239, 598, 4536, 4535, 2967, 1121, 1108, and 4531

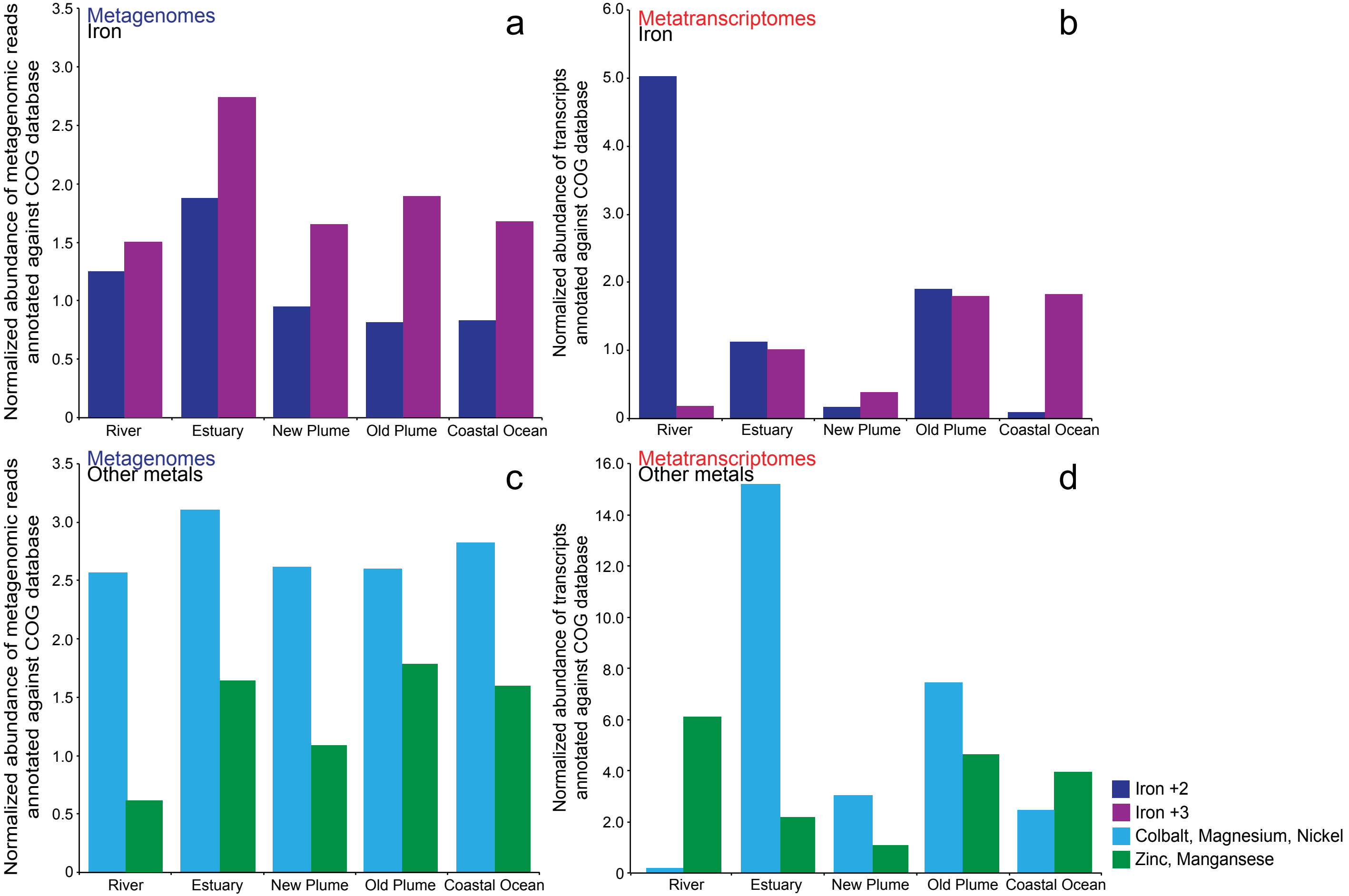

Supplement: S2 Fig — Abundance (a) and expression (b) of iron transporters, represented by COGs: 1840,1178, 614, 609, 1120, 1918, 370, 3470. Abundance (c) and expression (d) of cobalt, magnesium, nickel, zinc, and manganese transporters, represented by COGs: 5266, 310, 1122, 619, 2239, 598, 4536, 4535, 2967, 1121, 1108, and 4531. (PDF) [file pone.0140578.s002.pdf]
