# Supplementary material for: Microbial Gene Abundance and Expression Patterns across a River to Ocean Salinity Gradient
Source: PLoS One. 2015 Nov 4;10(11):e0140578. doi: 10.1371/journal.pone.0140578 (PMC4633275; doi:10.1371/journal.pone.0140578)

# S3 Fig. Manganese transporter gene abundance and expression.

Abundance (a) and expression (b) of two common manganese transporters, *mntH* and *sitABCD* across salinity.

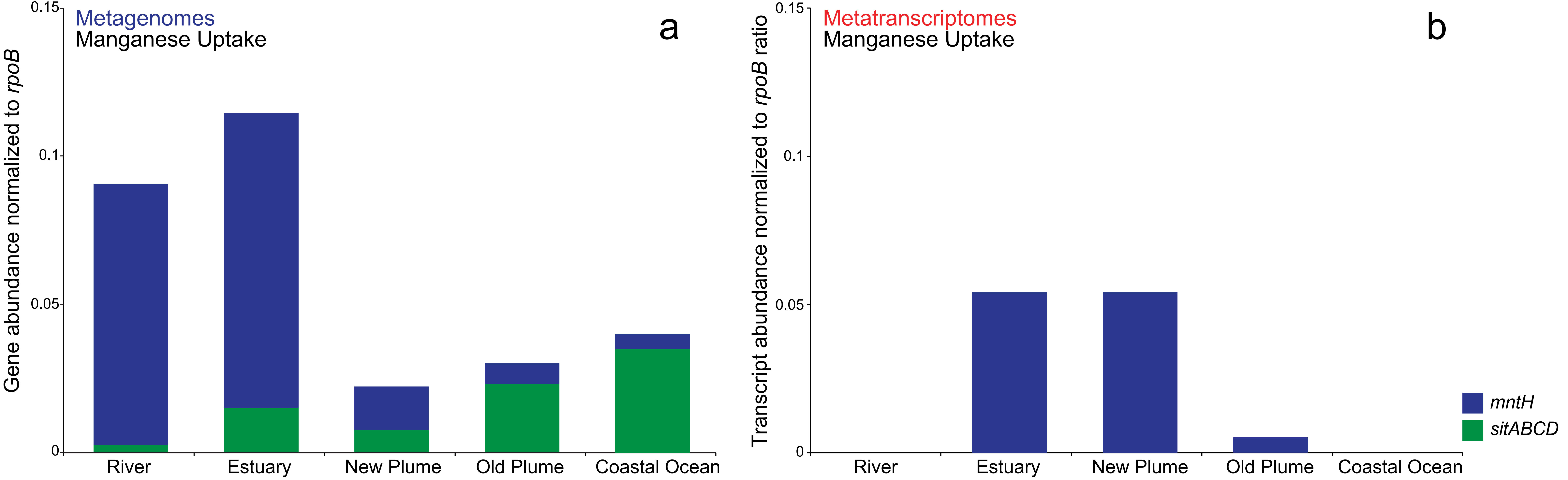

Supplement: S3 Fig — Abundance (a) and expression (b) of two common manganese transporters, mntH and sitABCD across salinity. (PDF) [file pone.0140578.s003.pdf]
